# Acquired pulmonary arteriovenous malformation associated with bronchiectasis: a case report

**DOI:** 10.1186/s13256-021-03233-2

**Published:** 2022-01-21

**Authors:** Yasutaka Kawasaki, Masafumi Nojiri, Taku Oikawa, Kazuaki Nishiki, Keisuke Nakase, Yutaka Takahara, Shiro Mizuno

**Affiliations:** 1 Department of Respiratory Medicine, Kanazawa Medical University, 1-1 Daigaku, Uchinada-machi, Kahoku-gun, Ishikawa, 920-0293 Japan; 2 Department of Internal Medicine, Wajima Municipal Hospital, 1-1 ha Yamagishi-machi, Wajima-shi, Ishikawa, 928-8585 Japan

**Keywords:** Pulmonary arteriovenous malformation, Bronchiectasis, Coil embolization

## Abstract

**Background:**

Pulmonary arteriovenous malformations are mostly caused by congenitally abnormal shunts between pulmonary arteries and pulmonary veins.

**Case presentation:**

A 74-year-old Japanese woman with a history of bronchiectasis was admitted to our hospital because of dyspnea on exertion. Pulmonary angiography and reconstructed three-dimensional contrast-enhanced computed tomography images showed shunts between pulmonary arteries and pulmonary veins, indicating a diagnosis of pulmonary arteriovenous malformations. Coil embolization of the shunts was successful.

**Conclusions:**

Our findings imply that bronchiectasis can cause pulmonary arteriovenous malformations, and thus patients who present with hypoxemia with bronchiectasis should be carefully evaluated.

## Background

Pulmonary arteriovenous malformations (PAVMs) are caused by abnormal shunts between pulmonary arteries and pulmonary veins without pulmonary capillaries, and can induce hypoxemia, cyanosis, and dyspnea. Most PAVMs are congenital, but they can also be acquired. We describe a patient with acquired PAVMs associated with bronchiectasis. Reconstructed three-dimensional (3D) contrast-enhanced computed tomography (CECT) images were useful for diagnosing this disorder.

## Case presentation

A 74-year-old Japanese woman presented with a 6-month history of gradually worsening dyspnea on exertion, a 10-year history of bronchiectasis, a 4-year history of infection with *Mycobacterium avium* complex, and pulmonary mucosa-associated lymphoid tissue (MALT) lymphoma in remission that had been treated by surgical resection of the right lower lobe and subsequent chemotherapy with single-agent rituximab. She had no history of recurrent epistaxis, and her family history did not include hereditary hemorrhagic telangiectasia (HHT) or PAVMs. She was admitted with pneumonia, symptoms of which were only partially resolved by antibiotic therapy as dyspnea on exertion persisted.

**Fig. 1:**
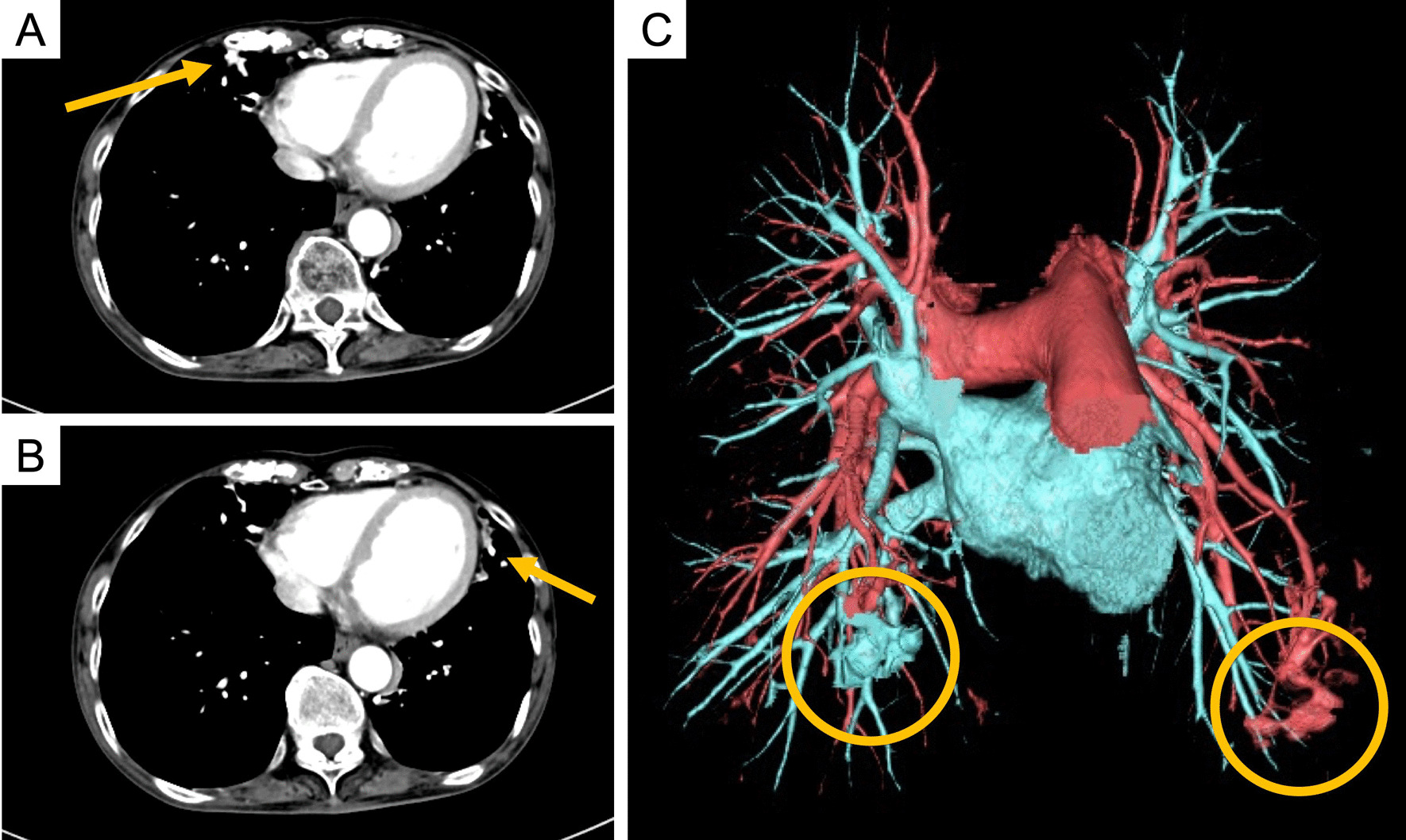
Findings of contrast-enhanced chest computed tomography and reconstructed 3D-contrast-enhanced computed tomography images. Contrast-enhanced chest computed tomography images show pulmonary veins of right middle lobe and left lingular segment in arterial phase (**A**, **B**; arrow). Reconstructed 3D-contrast-enhanced computed tomography image shows abnormal intrapulmonary shunts in right and left pulmonary artery and vein (**C**; circles)

Overall, physical examination findings were unremarkable. Her lungs were clear to auscultation, and clubbing or evidence of telangiectatic lesions on the nasal mucosa or skin was absent. Blood findings revealed C-reactive protein of 0.32 mg/dL and an erythrocyte sedimentation rate of 50 mm/hour. Arterial blood gas analysis showed slight hypoxemia (PaO_2_ of 65.9 mmHg on ambient air). Chest radiography showed increased density in the lower lung. Chest computed tomography (CT) showed bronchiectasis and increased density in the right middle lobe and the lingular segment of the left lung. Contrast-enhanced chest CT (Fig. 1) showed no evidence of pulmonary thromboembolism, but the pulmonary veins of the right middle lobe and left lingular segment were enhanced in the arterial phase. Reconstructed 3D-CECT images revealed abnormal intrapulmonary shunts in the right and left pulmonary arteries and veins. Echocardiography findings did not indicate vascular heart disease. Measuring PaO_2_ and SaO_2_ after breathing 100% oxygen for 20 minutes [1] revealed a shunt fraction of 22%. Whole-body ^99m^Tc-macroaggregated albumin (MAA) perfusion lung imaging revealed a shunt fraction of 21.3%. Head magnetic resonance imaging (MRI) findings were normal.

**Fig. 2:**
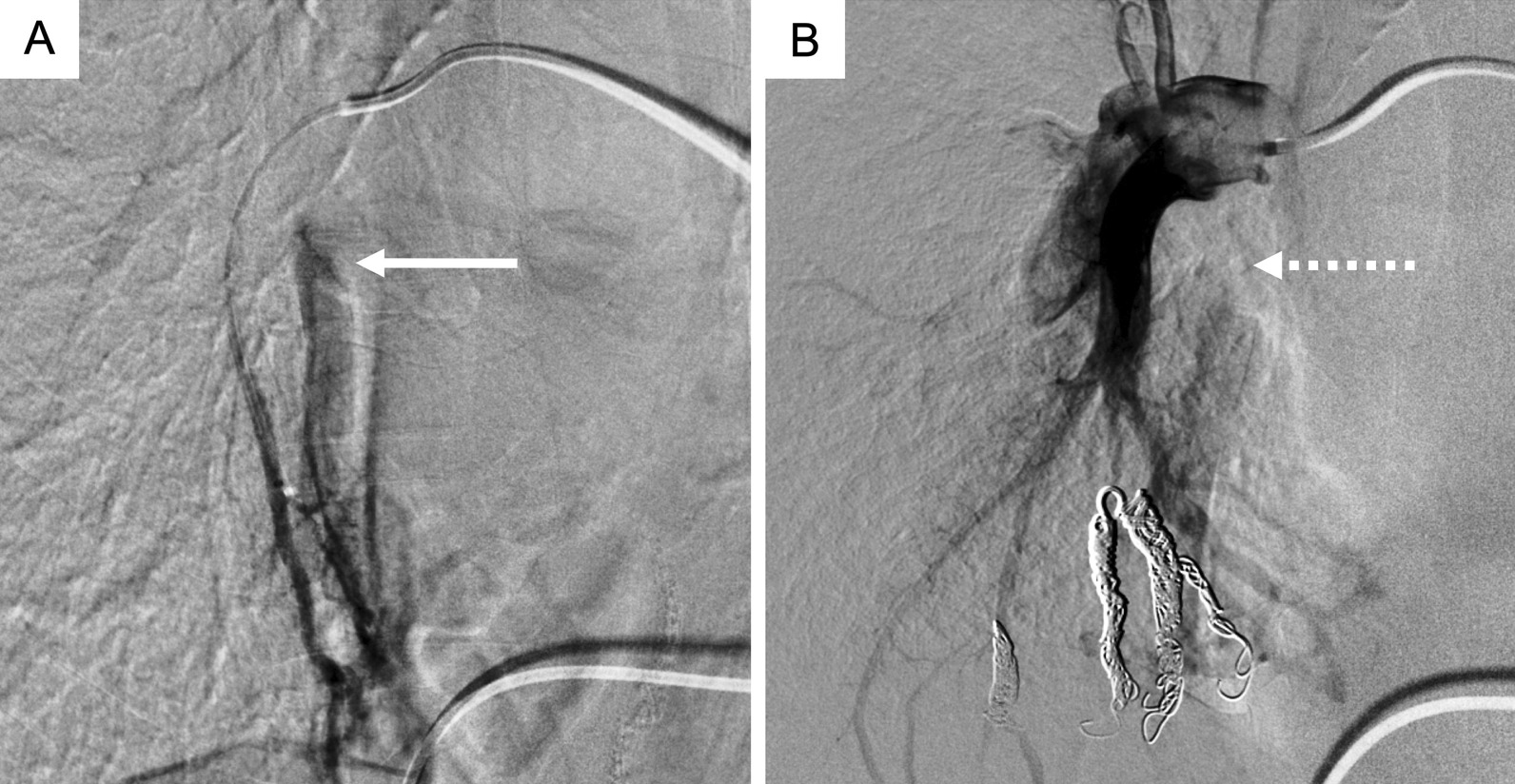
Pulmonary angiography findings. Images show irregular staining of pulmonary arteries in right middle lobe and pulmonary vein during early phase (**A**; arrow). Vein in early phase is not discernible after embolization (**B;** dotted arrow)

**Fig. 3:**
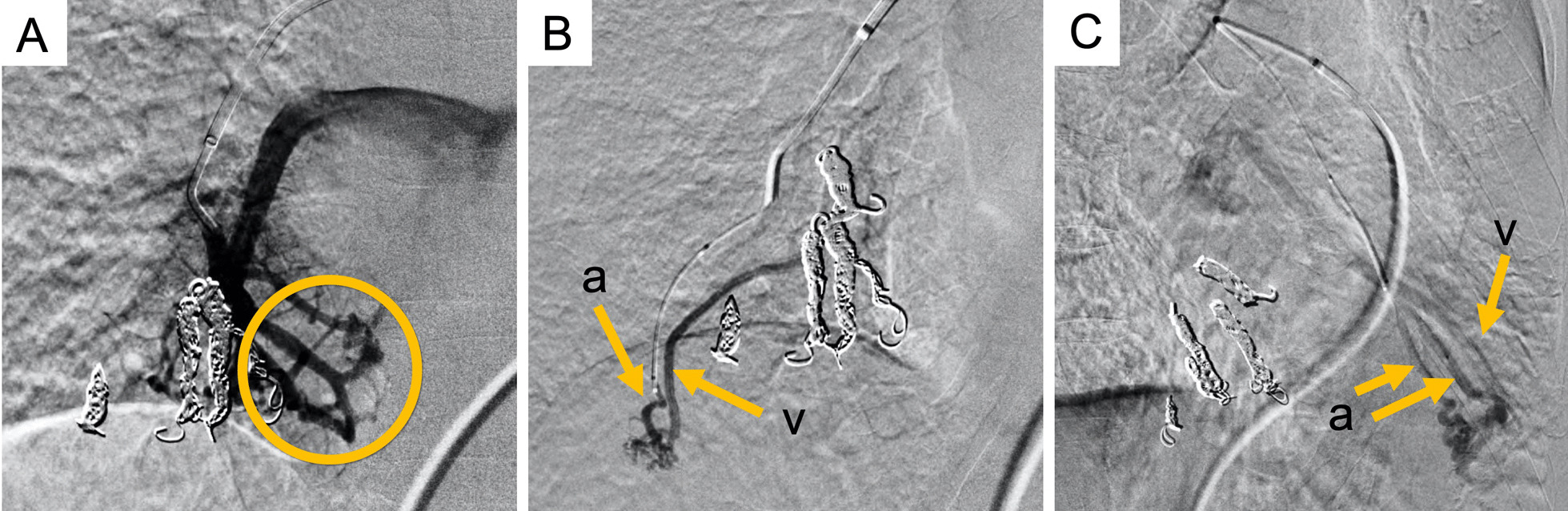
Pulmonary angiography findings at readmission. Images show abnormal shunt lesions in periphery of another area of right middle lobe (**A**; circle, **B**) and left lingular segment (**C**) *a* feeding arteries, *v* drainage veins

Pulmonary angiography revealed irregular staining of the pulmonary arteries in the right middle lobe and the pulmonary vein during the early phase (Fig. 2A). Although the typical sac and nidus of PAVMs were not evident, an abnormal shunt was suspected and treated by transcatheter coil embolization. Thereafter, the vein in the early phase was undetectable (Fig. 2B). The shunt fraction decreased to 12.4%, on ^99m^Tc-MAA lung perfusion images, and the dyspnea on exertion was ameliorated, so the patient was discharged. However, pneumonia reoccurred 3 months later and was improved with antibiotics, but hypoxemia persisted. The shunt fraction on ^99m^Tc-MAA lung perfusion images was elevated to 16%, indicating recanalization of the embolized shunts. Pulmonary angiography did not reveal evidence of recanalization, but new abnormal shunt lesions were found in the periphery of another area of the right middle lobe and in the left lingular segment (Fig. 3). The hypoxemia and dyspnea improved after coil embolization of these new lesions, and the patient was discharged. She has remained free of shunt recurrence and is presently under follow-up as an outpatient.

## Discussion and conclusions

Abnormal shunts between pulmonary arteries and pulmonary veins cause PAVMs, which are considered rare (prevalence 1 per 2600) [2].

Depending on the etiology, PAVMs are classified as congenital or acquired, and 60–90% are congenital manifestations of autosomal dominant syndrome HHT, also known as Osler–Weber–Rendu syndrome [3, 4]. Our patient did not have a history of recurrent epistaxis or telangiectasias, or family history of HHT; thus, she did not meet the Curaçao criteria [5]. Acquired PAVMs have been identified in established hepatic cirrhosis [6], and less frequently in trauma, schistosomiasis, actinomycosis [7], and metastatic thyroid carcinoma [8].

The precise pathogenesis of PAVMs is unknown. However, HHT can be caused by genetic mutations in the *ENG*, *ACVRL1*, and *MADH4* genes that encode proteins of the transforming growth factor-beta (TGF-β) superfamily [3, 4]. Dysfunctional TGF-β signaling causes abnormal capillary formation and maturation, leading to venous enlargement, vascular hyperbranching, and arteriovenous malformations, which explain the abnormal morphogenesis of vasculature in HTT [9]. The pathogenesis of acquired PAVMs with cirrhosis, hepatopulmonary syndrome, is considered to be mainly caused by abnormal pulmonary vasodilation resulting from elevated levels of nitric oxide (NO) and pulmonary angiogenesis resulting from the activation of vascular endothelial growth factor (VEGF) [10]. These findings suggest that PAVM development is associated with vascular dysplasia and abnormal angiogenesis. PAVM related to chronic infection has only been infrequently reported. Thomas *et al*. described a patient with PAVM possibly caused by tuberculous infection based on histopathological findings [11]. Abnormal shunts developed at sites of bronchiectasis in the middle lobe and lingula in our patient, indicating that abnormal angiogenesis due to chronic respiratory infection might have been involved in PAVM development.

CT scans undertaken while investigating for bronchiectasis could not have identified PAVMs. Nodules or mass-like circular or elliptical, defined, uniform shadows can be manifestations of PAVMs on plain CT. However, our patient had bronchiectasis at the right middle lobe and left lingular segment, precisely where PAVMs developed, thus causing difficulties with identifying PAVM shadows. We suspected PAVM from the unexplained dyspnea on the plain CT findings. We then further assessed the findings of reconstructed 3D-CECT imaging, which is useful for identifying abnormal pulmonary shunts and pulmonary vascular fistulas, and for evaluating feeding and drainage vessels.

Previously, pulmonary vascular shunts were surgically resected to treat PAVMs, whereas pulmonary vascular coil embolization is now the first choice of treatment due to advances in endovascular techniques [12, 13]. Our patient was treated by coil embolization without any complications, and oxygenation was regained, thus avoiding the need to inhale supplemental oxygen. However, she developed pneumonia and new shunt lesions that also required embolization. Her clinical condition has remained stable, but she is under follow-up to monitor recurrence and the further development of new pulmonary shunt lesions.

In summary, we describe a patient for whom reconstructed 3D-CECT images were helpful to conclude a diagnosis of acquired PAVMs associated with bronchiectasis. Some patients with bronchiectasis-associated PAVMs might be misdiagnosed, and a subset of patients might have “hidden” PAVMs. Therefore, hidden PAVMs should be considered when patients with bronchiectasis present with dyspnea that cannot be explained only by airway lesions. Reconstructed 3D-CECT images might be more widely applied to assess patients with hypoxemia on a background of bronchiectasis.

## Data Availability

Not applicable.

## Abbreviations

PAVMs: Pulmonary arteriovenous malformations
3D: Three-dimensional
CECT: Contrast-enhanced computed tomography
HHT: Hereditary hemorrhagic telangiectasia
CT: Computed tomography
MAA: Macroaggregated albumin
MRI: Magnetic resonance imaging
TGF-β: Transforming growth factor-beta
VEGF: Vascular endothelial growth factor
